# Original article: novelty of Canadian manufacture nasopharyngeal swabs for collection of samples being tested for SARS-CoV-2 in a pandemic setting

**DOI:** 10.3389/fpubh.2024.1344295

**Published:** 2024-05-09

**Authors:** Sandra Palomino-Padilla, Guillermo Caceres-Cardenas, Rodrigo Calderon, Alex C-T. Ko, Lauren Garnett, Kaylie Doan, Patrick Chong, Hammerly Lino, Tatiana Caceres, Teodor Veres, Claudia C. Dos Santos, Birgit Nielsen, Cesar Ugarte-Gil

**Affiliations:** ^1^Universidad Peruana Cayetano Heredia, Lima, Peru; ^2^Medical Devices Research Centre, National Research Council Canada (NRC), Boucherville, ON, Canada; ^3^National Microbiology Laboratory, Public Health Agency of Canada (PHAC), Winnipeg, ON, Canada; ^4^National Laboratory for HIV Immunology, Public Health Agency of Canada, Winnipeg, ON, Canada; ^5^National Research Council Canada (NRC), Ottawa, ON, Canada; ^6^University of Toronto, Toronto, ON, Canada

**Keywords:** swabs, nasopharyngeal, RT-PCR, SARS-CoV-2, COVID-19

## Abstract

**Objectives:**

The COVID-19 pandemic caused a global shortage of nasopharyngeal (NP) swabs, required for RT-PCR testing. Canadian manufacturers were contacted to share NP swab innovations. The primary objective was to determine whether novel NP test swabs were comparable to commercially available swabs regarding user characteristics, ability to collect a specimen, and diagnostic performance using RT-PCR testing.

**Methods:**

Participants were randomized by swab (test/control) and nostril (left/right). A calculated positive percent agreement ≥90% was considered successful. Mean Ct values of viral genes and housekeeping gene (RNase P) were considered similar if a Ct difference ≤ 2 between control and test group was obtained. There also was a qualitative assessment of swabs usability.

**Results:**

647 participants were enrolled from Huaycan Hospital in Lima, Peru, distributed over 8 NP swabs brands. Seven brands agreed to share their results. There were no statistically significant differences between the test swabs of these 7 brands and control swabs.

**Conclusion:**

All the seven brands are comparable to the commercially available flocked swabs used for SARS-CoV-2 regarding test results agreement, ability to collect a specimen, and user characteristics.

## Introduction

The world has gone through a crisis due to the coronavirus disease (COVID-19) pandemic caused by severe acute respiratory syndrome coronavirus 2 (SARS-CoV-2). By the end of January 2024, more than 703 million confirmed COVID-19 cases and more than 6.9 million deaths were reported worldwide (1). COVID-19 is a highly transmissible infection and it has a variety of signs and symptoms, including mild respiratory symptoms and fever (2). Many infected patients can be asymptomatic; however, they still have the potential to transmit the virus to other individuals (3). Therefore, the presence of symptoms alone is not a reliable way to determine who may be infected, who should be isolated, or who should be asked to isolate to prevent and control the transmission of the virus (4). Therefore, diagnostic testing is of utmost importance in any suspected case, whether symptomatic or not, to determine the presence of an infection.

In this scenario, testing for this virus using respiratory samples remains the only way to confirm infection. Molecular reverse transcription polymerase chain reaction (RT-PCR) is the standardized and recommended test worldwide for the diagnosis of SARS-CoV-2 infection, and despite the use of many samples of AN or MT swabs for diagnosis, at the time of the study, NP swabs showed to be the sample collection method with higher specificity and sensitivity (5).

At the beginning of the pandemic, the availability of SARS-CoV-2 testing was highly dependent on the availability of NP swabs, which was highly susceptible to the breakdown of the supply chain, during high-demand situations. The limited supply internationally led to scarcity that impacted health care globally, contributing to the accelerated spread of the virus (6). In Peru, at the time of the study, the demand for COVID-19 diagnostic tests was rapidly increasing with no tools that could meet the demand for proper epidemiological tracing and with a large population that could not have access to healthcare services because of the precarity of the health system, finding of new diagnostic alternatives was mandatory. During the development of this clinical trial, Peru was facing the second wave with an enormous number of patients who needed early detection of the disease, the government also required control over the number of new cases in record time, which was one of the main challenges that this study overcame. In this context, NP swabs using 3D printing and injection molding technology, represented an alternative that would allow for increased SARS-CoV-2 testing in a pandemic setting.

In May 2020, guidance (safety and effectiveness requirements for COVID-19 test swabs) was developed by Health Canada to support standardized testing of NP swabs in Canada. In addition, the COVID-19 Interim Order was published by Health Canada, under which the Minister of Health determined that there was an urgent public health need for medical devices used in the context of the COVID-19 pandemic (7). Before then, NP swabs were designated as a Class I medical device in Canada; therefore, manufacturers were generally not required to produce evidence of clinical effectiveness, such as clinical trials, clinical reviews, meta-analyses and real-world evidence reviews. Under the COVID-19 Interim Order, Health Canada established the safety and effectiveness requirements for novel NP swabs for SARS-CoV-2 (8). Requirements included quality manufacturing, design verification and validation, sterilization and packaging validation, and biocompatibility testing. Health Canada was one of the first regulatory bodies worldwide to have established quality criteria for NP swabs (8).

The final regulatory requirement to be met before the novel swabs could be authorized for sale in Canada was clinical testing for effectiveness in SARS-CoV-2-positive patients. To accelerate the commercialization of the swabs, the National Research Council of Canada Industrial Research Assistance Program (NRC IRAP) supported a comprehensive study to determine the clinical suitability of 3D printed NP swabs through collaboration with other Canadian governmental agencies, academic institutions, and clinical trial experts. This study is composed of two main phases, a pre-clinical testing phase and a clinical testing phase, the latter falling within the scope of the present paper. Pre-clinical testing included mechanical characterization, simulation testing using an airway trainer model, and *in vitro* testing of the test swabs at NRC’s Research Centres, St. Boniface Hospital, and the National Microbiology Laboratory of Canada, respectively. With positive pre-clinical results, the swab would proceed to clinical testing conducted with researchers at the Instituto de Medicina Tropical Alexander von Humboldt at Universidad Peruana Cayetano Heredia in Lima, Peru. MEDTEQ+, Montreal, coordinated the clinical testing in collaboration with Canadian swab manufacturers and with funding support from NRC IRAP. This collaboration supported and enhanced the possibilities of finding new alternatives for the early detection of COVID-19 preventing patients from being diagnosed in late stages that require hospitalizations and more complex clinical management in the context of a weak health system.

The primary objective of this study was to determine whether the newly designed and manufactured NP swabs (test swabs) were comparable to commercially available flocked swabs concerning user characteristics, ability to collect a specimen, and test results agreement.

Using a novel testing framework and molecular RT-PCR testing, this study tested the safety and effectiveness of novel NP swabs relative to existing commercially available flocked swabs. This would increase the availability of NP swabs for COVID-19 testing for the vast majority of the population and thereby help identify cases and subsequently reduce the transmission of the virus, which was a major concern for adequate control of the spread of infection.

## Methods

The clinical trial for medical devices used a single-arm design in which the patients under investigation (PUIs) were not compared with any other cohort. The enrollment of participants was conducted in Perú, from January to July 2021, at the Hospital de Huaycán located in Lima, Perú. Adults who presented to the healthcare center and met all the enrollment criteria were asked to participate in the study. The clinical protocol was approved by the Institutional Ethics Committee in Investigation, of the Universidad Peruana Cayetano Heredia (SIDISI 203607) and by the National Research Council of Canada’s Research Ethics Board, and was registered in the PRISA repository for clinical research studies from the Peruvian National Institute of Health (EI00000001698). Results are reported as per Strengthening the Reporting of Observational Studies in Epidemiology (STROBE) guidelines.

### Inclusion criteria

Adults 18 years of age or older; inpatient or outpatient presenting to a SARS-CoV-2 testing center for testing; two days or more since the onset of symptoms, met the WHO definition criteria for a suspected COVID-19 case (9); and be willing and able to give informed consent (Supplementary Figure S1A). Alternatively, the participant could not be enrolled in the study if any of the exclusion criteria were met, including bleeding disorder and/or low platelet count (thrombocytopenia of <50,000 platelets/μL); taking a systemic anticoagulant; and/or deviated septum significant enough to prevent insertion of the NP swab into each of the nostrils.

### Sample collection

PUIs that consented to the study were swabbed according to local standard practice, and the procedural order was determined through randomization. PUIs were swabbed with a commercially available flocked swab in one nostril and with the test swab in the opposite nostril for sample specimen collection. The order in which the swabs were inserted into the nostril could have potentially impacted the results of the test because of the potential increased mucus in the area caused by tearing eyes and local irritation that may have been experienced while the procedure was being performed. Therefore, the order in which the NP swabs were inserted into the left or right nostril (either test or control swab) was randomized. This study compared a total of 8 NP test swabs, with 7 companies agreeing to include their results in this publication. In no specific order, the swab results included in this paper are from PAMA (SWB-0007), Papp Plastics (PNNS-1), Mitchell Plastics (MP0010), Southmedic (SwabMedic CSWB-01), Tronosjet (JetSwab), PriMed (NPSC04) and Canadian Hospital Specialties (BXSWAB-3DHP). An overview of the swabs is provided in Table 1. These swabs were compared to one brand of commercially available NP swab (Norgen iClean Swab^®^ part number CM-96000), which was used as a control swab (standard of reference device). Each brand concluded its evaluation when 30 PUIs tested positive for SARS-CoV-2 using the reference control swab.

**Table 1:** Nasopharyngeal swab manufacturing details.

Swab manufacturer	Model	Manufacturing method	Photo
Norgen iClean	CM 96000	Flocked	
PAMA Manufacturing	SWB-0007	3D printed	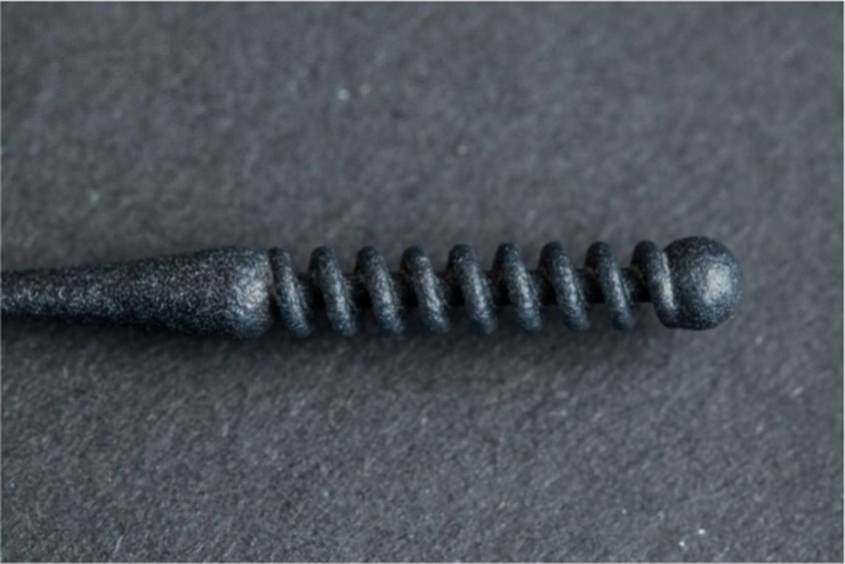
Papp Plastics & Distributing Ltd	PNNS-1	Injection molded	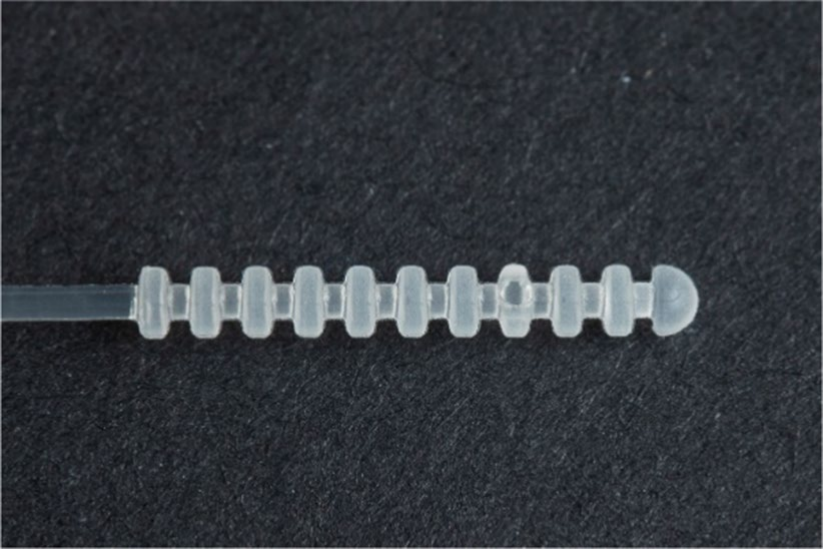
Mitchell Plastics Inc.	MP0010	Injection molded	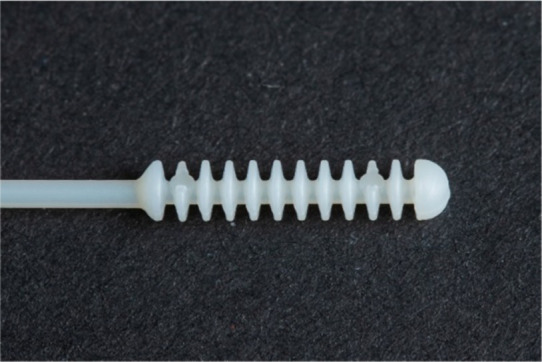
Southmedic Inc.	CSWB-01	Injection molded	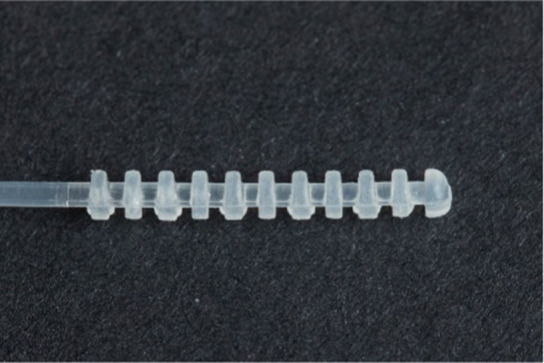
Tronosjet Maintenance Inc.	JetSwab	3D printed	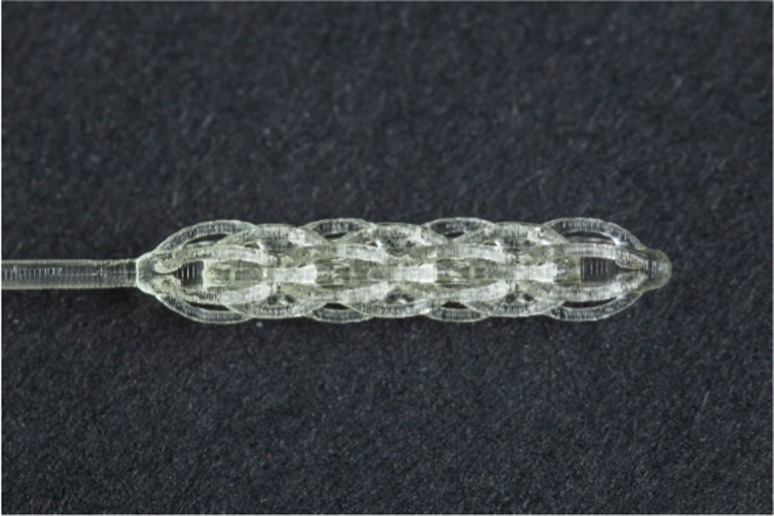
Canadian Hospital Specialties Ltd.	BXSWAB-3DHP	3D printed	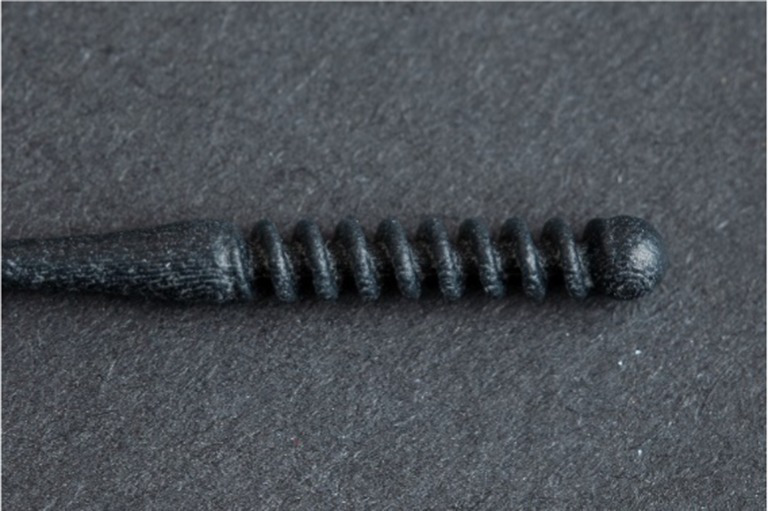
PriMed Instruments Inc.	NPSC04	Bristle	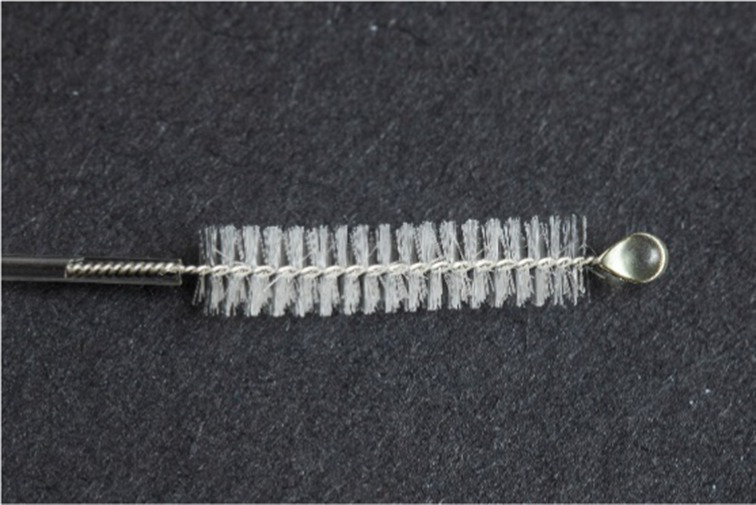

In either case, a trained healthcare provider inserted the NP swab into a nostril, rubbed it in a circle around the nostril five times, removed it, and broke off the tip into a vial containing VTM. The procedure was repeated in the opposite nostril with the remaining NP swab (either test or control swab based on randomization) with the same procedure being performed for each. The vials containing VTM (Puritan^®^ UniTranz-RT Transport Systems) were pre-labeled, and the healthcare provider remained unblinded for the entirety of the procedure. The VTM and tubes used in the trial had been validated by the National Microbiology Lab, Winnipeg, to ensure that they did not interfere with RT-PCR results.

### Transport and processing of the samples

Collected specimens were immediately placed in coolers, stored between 2 to 8°C, and transported to the Instituto de Medicina Tropical Alexander von Humboldt at Universidad Peruana Cayetano Heredia. An aliquot of VTM from both vials was taken and used for molecular RT-PCR testing as per the testing protocol. RNA extraction was performed using Qiagen viral RNA extraction Kit. To amplify viral RNA, 8uL aliquots were run on BIO-RAD (CFX96 Real-Time System) using Norgen’s 2019-nCoV TaqMan RT-PCR Kit. Inconclusive results were tested twice by RT-PCR, and discordant results were reported in the corresponding CRF.

The primary objective of this study was to determine whether the newly designed and manufactured NP swabs (test swabs) were comparable to commercially available flocked swabs (Norgen iClean) for user characteristics, ability to collect a specimen, and test results agreement. Also, the objective was to assess diagnostic RT-PCR test performance agreement, the ability to collect a specimen, and the usability using qualitative assessments (i.e., flexibility, fit, and ability to navigate to the nasopharynx).

### Statistical analysis

Demographic data was summarized using descriptive statistics. Descriptive statistics were presented as number and relative frequency (%) for categorical variables and number, mean ± standard deviation (SD) for numerical variables. The positive percent agreement, negative percent agreement, and associated 95% confidence interval (CI) were calculated to assess diagnostic performance in comparison to results obtained from commercially available flocked swabs (Norgen iClean). A calculated positive percent agreement ≥90% was considered a success.

Ct values were compared between the test NP swab and control NP swab for all patients who tested positive for SARS-CoV-2 on the control swab using a paired analysis approach performed separately for both the target SARS-CoV-2 viral gene and the housekeeping gene (RNase P). The comparison was based on the cases where both the test and the control swabs were positive and reported as valid results. The test and control NP swabs were considered similar in their ability to pick up a sample if the absolute RNase P mean Ct difference was ≤2.

The usability analysis compared flexibility, fit, and ability to navigate the nasopharynx of both the test and control swabs. A comparison table was used to compare the test NP swab to the control NP swab, using Yes/No answers plus descriptors. All statistical analyses were performed using R version 4.0.2.

## Results

A total of 647 participants were enrolled and distributed over 8 different NP swab test brands; however, only 7 brands (n = 549) agreed to share their results in this paper. Demographic characteristics are available in Table 2.

**Table 2:** Demographic characteristics of the study population.

	Brand A	Brand B	Brand C	Brand D	Brand E	Brand F	Brand G
Demographic characteristics	*N* = 86	*N* = 60	*N* = 107	*N* = 62	*N* = 101	*N* = 71	*N* = 62
Age (years)							
Mean (SD)	37 (12)	38 (13)	35 (13)	38 (14)	35 (15)	38 (15)	39 (12)
Median (IQR)	36 (29, 45)	37 (25, 44)	34 (25, 45)	34 (27, 48)	32 (23, 45)	35 (25, 46)	38 (30,47)
Range	18, 64	18, 75	18, 77	18, 84	18, 80	18, 75	18,67
Sex							
Male	42/86 (49%)	28/60 (46.7%)	40/107 (37.4%)	32/62 (52.0%)	43/101 (42.5%)	26/71 (36.6%)	21/62 (34.0%)
Female	44/86 (51%)	32/60 (53.3%)	67/107 (62.6%)	30/62 (48.0%)	58/101 (57.4%)	45/71 (63.4%)	41/62 (66.0%)
Participant recruitment							
Inpatient	0/86 (0%)	0/60 (0.0%)	0/107 (0.0%)	0/62 (0.0%)	0/101 (0.0%)	0/71 (0.0%)	0/62 (0.0%)
Outpatient	86/86 (100%)	60/60 (100.0%)	107/107 (100.0%)	62/62 (100.0%)	101/101 (100.0%)	71/71 (100.0%)	62/62 (100.0%)

IQR: interquartile range, SD: standard deviation.

The mean days from onset of symptoms was as follows: 6.2 (Brand A), 5.1 (Brand B), 5.1 (Brand C) 4.9 (Brand D), 4.7 (Brand E), 4.7 (Brand F) and 5.6 (Brand G). The agreement percentage for each Brand from A to G was: 93.9, 96.7, 93.5, 93.3, 96.7, 88.2 and 100%, respectively.

Despite following the same instructions and procedures for each brand, for Brand F, data suggested an agreement percentage of 88.20%, which was below the approved percentage agreement (90%). In this group, eight control swabs were inconclusive, from which 7 (87.5%) were positive and 1 (12.5%) was inconclusive for the test swab. Inconclusive results were defined by laboratory values previously established in the product insert of Norgen’s 2019-nCoV TaqMan RT-PCR Kit, these values are standardized for laboratories around the world that were using this RT-PCR kit for COVID-19 detection. For a proper interpretation, Norgen’s 2019- nCoV TaqMan RT-PCR kit, has a positive and negative control, the negative control must be negative and not exhibit fluorescence growth curves that cross the threshold line. If there is any amplification with the negative control the run is not valid and the assay has to be repeated, in the same line the N/ORF1ab/RP positive control reaction should produce a positive result with an expected Ct value (<40.oo Ct) for each target, if the positive control does not provide a positive result the run is not valid and no interpretation of SARS-CoV-2 detection can be made. If both of them are exhibiting the correct results, and the N gene results differ from the ORF1ab results corresponding to the positive control, the result has to be interpreted as inconclusive.

However, when all the inconclusive results were excluded, Brand F’s NP swabs passed with a positive percent agreement of 93.70%. Also, for the thirty swabs with concordant positive results, the mean Ct value was 25.8 (7.92) for the control swab and 23.9 (7.74) for the test swab with a difference of 1.9 [95% CI 0.7, 3.2], and the mean Ct value for the RNase P was 27.8 (2.50) for the control swab and 25.8 (1.42) for the test swab with a difference of 1.9 [95% CI 1.2, 2.6].

The specific data of positive and negative results, Ct values and RNase *p* values for each brand are described in Table 3. Regarding the usability analysis, which compares flexibility, fit and ability to navigate to the nasopharynx of both test and control swabs, there were no major differences nor issues between the test brands compared to the control (Table 4).

**Table 3:** Test performance agreement results by brand.

		Brand A	Brand B	Brand C	Brand D	Brand E	Brand F	Brand G
Mean onset of symptoms		6.2 (SD 2.8)	5.1 (SD 7.21)	5.1 (SD 1.67)	4.9 (SD 1.73)	4.7 (SD 1.32)	4.7 (SD 1.28)	5.6 (SD 2.15)
N° positive control swab result		33	30	31	30	30	34	33
	Positive test swab	31 (94.0%)	29 (96.7%)	29 (93.5%)	28 (93.0%)	29 (96.7%)	30 (88.2%)	33 (100.0%)
	Negative test swab	2 (6.1%)	1 (3.3%)	0 (0,0%)	1 (3.3%)	0 (0.0%)	2 (5.9%)	0 (0.0%)
	Inconclusive test swab	0 (0.0%)	0 (0.0%)	2 (6.5%)	1 (3.3%)	1 (3.3%)	2 (5.9%)	0 (0.0%)
Agreement percentage (%)		93.9%	96.7%	93.5%	93.3%	96.7%	88.7%	100.0%
Concordant positive test and control swab		31 (94.0%)	29 (96.7%)	29 (93.5%)	28 (93.0%)	29 (96.7%)	30 (88.2%)	33 (100.0%)
Control swab	Mean Ct	23.3 (SD 4.7)	22.9 (SD 6.18)	23.7 (SD 7.46)	22.2 (SD 6.09)	23.7 (SD 6.84)	25.8 (SD 7.92)	22.0 (SD 5.54)
	RNAse P	27.8 (SD 3.6)	28.1 (SD 3.48)	27.0 (SD 2.29)	28.7 (SD 1.45)	28.4 (SD 1.72)	27.8 (SD 2.50)	29–0 (SD 1.89)
Test swab	Mean Ct	23.4 (SD 4.9)	22.0 (SD 5.36)	24.7 (SD 7.80)	22.8 (SD 5.20)	23.8 (SD 7.19)	23.9 (SD 7.74)	22.6 (SD 6.26)
	RNAse P	29.3 (SD 3.2)	27.3 (SD 2.36)	27.4 (SD 2.89)	27.7 (SD 1.89)	27.3 (SD 1.23)	25.8 (SD 1.42)	29.0 (SD 2.0 6)

Ct: cycle threshold, SD: standard deviation.

**Table 4:** Qualitative assessment.

	Brand A	Brand B	Brand C	Brand D	Brand E	Brand F	Brand G
	*N* = 86*	*N* = 60*	*N* = 107*	*N* = 62*	*N* = 101*	*N* = 71*	*N* = 62*
Comparable flexibility°							
No	0/86 (0.0%)	2/60 (3.3%)	1/107 (0.9%)	1/62 (1.6%)	1/101 (1.0%)	0/71 (0.0%)	0/62 (0.0%)
Yes	86/86 (100.0%)	58/60 (96.7%)	106/107 (99.1%)	61/62 (98.4%)	100/101 (99.0%)	71/71 (100.0%)	62/62 (100%)
Comparable fit°							
No	1/86 (1.2%)	0/60 (0.0%)	0/107 (0.0%)	1/62 (1.6%)	0/101 (0.0%)	0/71 (0.0%)	0/62 (0.0%)
Yes	85/86 (99%)	60/60 (100.0%)	107/107 (100.0%)	61/62 (98.4%)	101/101 (100.0%)	71/71 (100.0%)	62/62 (100%)
Comparable nasopharynx navigation°							
No	1/86 (1.2%)	0/60 (0.0%)	0/107 (0.0%)	1/62 (1.6%)	4/101 (4.0%)	0/71 (0.0%)	0/62 (0.0%)
Yes	85/86 (99%)	60/60 (100.0%)	107/107 (100.0%)	61/62 (98.4%)	97/101 (96.0%)	71/71 (100.0%)	62/62 (100%)

* Sample size of all participants from each brand evaluated, including participants with positive, negative, inconclusive, or invalid results. °Questions answered by nurses regarding: flexibility, fit, and NP Navigation (i.e., Is the flexibility/fit/NP navigation of the test swab comparable to the reference swab?). NP: Nasopharyngeal.

## Discussion

In this study, seven different test brands had a positive percent agreement ≥90% and similar usability outcomes. Although some of the test swabs differed slightly in mean Ct values, none of them fell below the pre-specified threshold of a difference ≥ 2 Ct. This renders them very similar, as confirmed by the overall accuracy of RT-PCR testing, which is highly sensitive to small amounts of material (10).

RT-PCR continues to be one of the most accurate diagnostic methods available for the detection of SARS-CoV-2, which is fundamental for epidemiological surveillance and contact tracing; however, there could be several reasons why its sensitivity may be jeopardized. A meta-analysis that studied the accuracy of RT-PCR for COVID-19 detection suggested a pooled sensitivity of 89%, with several variations in sensibility values of different studies ranging from 50 to 100% (10). The possible main factors for these differences in sensibility may be related to the collection technique, viral load in the nasopharynx, and laboratory assays used for testing. Other studies reported that the main limitations of this diagnostic method are sampling time, type of specimen, viral kinetics, transport and storage conditions, and post-analytical variables (11). To eliminate any issues with the listed limitations, the above variables of concern remained constant throughout this study so that we could focus on the effect of changing the swab material component.

The quality of NP swabs is essential for test accuracy in collecting samples. Swab head structure and fibers are in direct contact with the organisms collected from the sampling site; thereby making the structure of the swabs critical for obtaining a precise diagnosis (12). Therefore, any change in the swab design might compromise its diagnostic ability. By comparing the diagnostic performance of commercially available swabs with newly 3D printed swabs, injection molded swabs, and a bristle-made swab, it may be possible to evaluate which swab structure is more suitable for diagnostic and clinical purposes. In our study, this evaluation demonstrated a high percentage of positive agreement between the control and test swabs, suggesting that the new swab designs which have many similarities to control swabs in terms of structure, length, and flexibility, are good candidates for closing the gap in the PCR-testing supplies shortage.

### Inconclusive test results

Some issues of the present study were the inconclusive PCR results that were seen in one specific brand, although the data collected related to this brand is not enough to establish a relationship between inconclusive results and a specific variable. Previous studies suggest that most inconclusive results arise when patients test after more than 15 days of symptoms, are asymptomatic, or have a previously confirmed infection with COVID-19. Also, RT-PCR assays are known to be affected by the type of sampling (NP/OP swab), RNA extraction methods, and lot-to-lot variation in RT-PCR kits (13).

One last limitation may be the clinical sensitivity of NP swabs, which some estimates can go as low as 70% (14). This could be due to sample collection and handling rather than a misdiagnosis by RT-PCR. Many variables can interfere with the overall performance of a test, which could include inappropriate handling during sample collection, transport, and processing, among others (15), which sometimes are variables that can be difficult to control and follow and that may have explained the variations between the first result of control swabs and the repeated results.

The main concern of the inconclusive results for the study was to determine if it was related to a specific brand and this may diminish the credibility of the analysis regarding the efficacy of the other brands that met the requirements previously established to be considered as effective as the standard swabs. That was the main reason to strengthen all the other variables that did not depend on factors related to the NP swab.

To date, many evaluations of medical devices have been restricted to a small number of participants; however, this study presents an evaluation of several brands in a large symptomatic population with similar days from the onset of symptoms, being this a strength of the study. Also, all of the inconclusive results were tested twice by RT-PCR, to reassure that its analytical sensitivity is proficiently related to the NP swabs clinical sensitivity, which could be more variable. Another strength of this study is that it includes a qualitative evaluation of swab characteristics and performance from the participant and study nurse’s point of view.

Finally, our results are in accordance with previous studies that also used 3D printing for swab elaboration. Additional studies have also evaluated the possibility of on-site, on-demand 3D-printed swabs inside hospitals or clinics, likewise showing positive results and acceptance (16–19).

In a pandemic context with supplies-chain issues (specifically for testing supplies), these results provide evidence of different available swabs as an adequate and qualified tool to perform the collection of nasopharyngeal samples and may represent a solution to the critical COVID-19 testing bottleneck, caused by the low availability of standard swabs.

In this study, mostly all the brands met the requirement to prove their efficacy compared with the standard NP swabs with a high agreement percentage, in a context where all the limitations commonly related to the collection and processing of samples were closely followed. Despite inconclusive results for one of the brands that could be explained by several factors such as transport or processing of samples, the study has strengths that have to be pointed out such as a high number of participants, the standardization of clinical criteria and the results for other 7 brands that showed good performance.

## Conclusion

In conclusion, our data indicates that all the seven brands reported in this paper, are comparable to the commercially available flocked swabs used for SARS-CoV-2 regarding test results agreement, ability to collect a specimen, and user characteristics. This shows that these swabs are an effective clinical device that can help solve the global shortage of this important diagnostic tool in a critical COVID-19 pandemic situation and will be useful in assisting future outbreaks of several respiratory diseases that can lead to a shortage of critical diagnostic materials.

## Data availability statement

The datasets presented in this article are not readily available because the raw data supporting the conclusions of this article will be made available by the corresponding author, upon reasonable request. Requests to access the datasets should be directed to CU-G cesar.ugarte@upch.pe.

## Ethics statement

The studies involving humans were approved by Comité Institucional de Ética de la Universidad Peruana Cayetano Heredia. The studies were conducted in accordance with the local legislation and institutional requirements. The participants provided their written informed consent to participate in this study.

## Author contributions

SP-P: Investigation, Writing – original draft, Writing – review & editing. GC-C: Investigation, Writing – original draft, Writing – review & editing. RC: Formal Analysis, Investigation, Writing – review & editing. AC-TK: Investigation, Supervision, Writing – review & editing. LG: Investigation, Methodology, Writing – review & editing. KD: Investigation, Methodology, Writing – review & editing. PC: Investigation, Methodology, Writing – review & editing. HL: Investigation, Methodology, Writing – review & editing. TC: Investigation, Methodology, Writing – review & editing. TV: Investigation, Methodology, Writing – review & editing. CD: Conceptualization, Investigation, Methodology, Writing – review & editing. BN: Conceptualization, Funding acquisition, Project administration, Supervision, Writing – review & editing. CU-G: Conceptualización, Data curation, Funding acquisition, Investigation, Methodology, Project administration, Writing – review & editing.
