# Supplementary material for: Original article: novelty of Canadian manufacture nasopharyngeal swabs for collection of samples being tested for SARS-CoV-2 in a pandemic setting
Source: Front Public Health. 2024 May 9;12:1344295. doi: 10.3389/fpubh.2024.1344295 (PMC11111943; doi:10.3389/fpubh.2024.1344295)
Supplement: Supplementary file 1 [file Data_Sheet_1.PDF]

Brand A

CODE	Control swab	Test swab	C			T		
			N1	N2	RnaseP	N1	N2	RnaseP
CSW0001	POSITIVE	POSITIVE	37.19	34.73	30.82	35.86	33.77	31.73
CSW0006	POSITIVE	POSITIVE	28.06	27.57	28	26.20	25.43	28.15
CSW0007	POSITIVE	POSITIVE	19.65	19.02	27.47	21.39	20.11	26.83
CSW0009	POSITIVE	POSITIVE	20.89	19.37	27.2	18.01	16.80	25.07
CSW0011	POSITIVE	NEGATIVE	39.30	34.76	27.56	N/A	N/A	29.03
CSW0012	POSITIVE	POSITIVE	30.81	32.30	28.14	24.37	24.78	25.66
CSW0022	POSITIVE	NEGATIVE	38.21	34.04	29.43	N/A	N/A	29.78
CSW0028	POSITIVE	POSITIVE	25.19	25.34	32.75	23.56	23.32	32.52
CSW0029	POSITIVE	POSITIVE	23.96	24.11	29.68	28.26	28.50	31.53
CSW0030	POSITIVE	POSITIVE	26.18	25.18	29.09	24.03	23.15	30.05
CSW0033	POSITIVE	POSITIVE	22.23	21.74	28.48	23.85	23.66	30.06
CSW0034	POSITIVE	POSITIVE	20.84	19.84	29.17	22.84	21.77	30.88
CSW0035	POSITIVE	POSITIVE	21.45	20.52	28.29	22.73	22.05	30.22
CSW0036	POSITIVE	POSITIVE	24.75	23.42	27.75	23.89	22.59	32.77
CSW0038	POSITIVE	POSITIVE	19.09	18.34	29.31	18.77	18.00	29.59
CSW0042	POSITIVE	POSITIVE	19.89	18.58	29.27	21.39	20.72	31.54
CSW0044	POSITIVE	POSITIVE	24.30	23.41	30.05	23.40	22.33	29.06
CSW0046	POSITIVE	POSITIVE	28,77	31,51	14.47	33.09	32.35	30.24
CSW0047	POSITIVE	POSITIVE	29,25	28,29	14.47	17,91	19,03	14.47
CSW0048	POSITIVE	NEGATIVE	35.68	35.37	31.13	N/A	N/A	30.5
CSW0049	POSITIVE	POSITIVE	22.65	21.87	29.29	22.97	22.47	33.33
CSW0053	POSITIVE	POSITIVE	16.28	16.55	27.7	18.91	19.09	30.4
CSW0056	POSITIVE	POSITIVE	22.47	21.38	28.42	22.11	21.20	31
CSW0057	POSITIVE	POSITIVE	16.31	15.24	28.47	16.65	15.93	30.18
CSW0062	POSITIVE	POSITIVE	26.09	26.30	29.32	27.75	27.90	30.57
CSW0063	POSITIVE	POSITIVE	18.96	19.17	28.11	18.10	18.19	29.78
CSW0066	POSITIVE	POSITIVE	16.18	15.37	28.62	20.27	19.53	31.48
CSW0070	POSITIVE	POSITIVE	23.39	22.79	28.35	25.42	24.22	29.05

CSW0072	POSITIVE	POSITIVE	22.61	22.85	25.66	34.44	33.69	27.96
CSW0073	POSITIVE	POSITIVE	28.69	27.71	29.17	16.93	16.02	28.43
CSW0077	POSITIVE	POSITIVE	22.21	21.46	27.83	25.28	24.64	28.52
CSW0079	POSITIVE	POSITIVE	20.88	19.93	27.41	21.90	20.87	29.05
CSW0082	POSITIVE	POSITIVE	27.26	26.55	29.34	31.95	31.36	30.16
CSW0085	POSITIVE	POSITIVE	22.65	22.04	28	21.95	20.70	27.9

Brand B

CODE	Control swab	Test swab	C			T		
			N1	N2	RnaseP	N1	N2	RnaseP
PAP0001	POSITIVE	POSITIVE	20.28	19.20	28.1	22.98	21.47	29.68
PAP0002	NEGATIVE	INCONCLUSIVE	N/A	N/A	30.46	N/A	34.47	26.78
PAP0003	INCONCLUSIVE	INCONCLUSIVE	N/A	39.26	27.69	N/A	35.6	28.72
PAP0004	INCONCLUSIVE	NEGATIVE	N/A	39.24	28.79	N/A	N/A	25.31
PAP0005	POSITIVE	POSITIVE	27.13	27.28	28.74	26.16	26.16	26.85
PAP0007	POSITIVE	INCONCLUSIVE	31.15	31.25	29.91	N/A	35.11	28.81
PAP0008	POSITIVE	POSITIVE	17.54	15.77	27.34	23.08	21.72	26.77
PAP0009	INCONCLUSIVE	POSITIVE	N/A	38.05	30.14	32.57	31.94	26.76
PAP0010	INCONCLUSIVE	NEGATIVE	N/A	33.93	28.06	N/A	N/A	28.12
PAP0012	POSITIVE	POSITIVE	30.08	30.04	29.38	31.03	30.72	27.56
PAP0013	POSITIVE	POSITIVE	33.68	29.85	29.22	26.13	25.22	27.19
PAP0015	POSITIVE	POSITIVE	23.72	22.98	29.97	25.13	24.15	29.77
PAP0016	POSITIVE	POSITIVE	22.07	21.12	27.07	23.88	22.45	27.66
PAP0017	POSITIVE	POSITIVE	N/A	42.2	30.49	N/A	N/A	28.55
PAP0019	POSITIVE	POSITIVE	23.28	22.1	29.02	19.9	18.42	26.67
PAP0020	POSITIVE	POSITIVE	23.09	22.24	28.73	25.19	24.59	29
PAP0021	POSITIVE	POSITIVE	18.31	17.32	30.4	13.58	12.24	26.8
PAP0023	POSITIVE	POSITIVE	38.04	32.61	31.41	19.78	18.33	28.35
PAP0025	POSITIVE	POSITIVE	22.51	21.4	27.92	24.57	23.81	26.47
PAP0026	POSITIVE	POSITIVE	20.51	19.12	28.3	21.59	20.32	26.92
PAP0028	POSITIVE	POSITIVE	23.18	23.42	27.17	21.03	20.79	26.02

PAP0030	POSITIVE	POSITIVE	17.84	16.56	27.17	15.9	14.54	25.98
PAP0031	POSITIVE	POSITIVE	19.76	18.77	27.95	19.89	19.1	29.03
PAP0032	POSITIVE	POSITIVE	23.37	22.5	30.4	20.11	18.4	26.63
PAP0033	POSITIVE	POSITIVE	33.59	32.34	28.65	20.62	19.15	26.12
PAP0034	POSITIVE	INCONCLUSIVE	N/A	N/A	29.93	N/A	36.76	26.54
PAP0036	NEGATIVE	POSITIVE	N/A	40.09	29.92	34.59	33.42	26.91
PAP0037	NEGATIVE	POSITIVE	N/A	N/A	27.18	38.93	35.78	27.66
PAP0038	POSITIVE	POSITIVE	14.16	13.09	26.41	16.68	14.94	26.82
PAP0039	POSITIVE	NEGATIVE	39.81	35.03	35.03	N/A	N/A	N/A
PAP0040	POSITIVE	POSITIVE	28.04	29.65	29.65	32.32	29.64	29.64
PAP0041	POSITIVE	POSITIVE	19.29	17.95	17.95	19.03	17.77	17.77
PAP0042	POSITIVE	POSITIVE	16.29	15.13	15.13	19.25	17.34	27.08
PAP0043	POSITIVE	POSITIVE	18.24	17.21	27.57	18.56	16.73	25.37
PAP0044	POSITIVE	POSITIVE	17.46	16.1	28.42	17.04	15.69	27.19
PAP0045	POSITIVE	POSITIVE	35.89	35.7	33.56	35.52	35.1	33.09
PAP0046	POSITIVE	POSITIVE	23.75	22.26	30.66	28.03	27.51	30.25
PAP0047	INCONCLUSO	POSITIVE	N/A	35.41	25.89	34.03	33.75	27.58
PAP0049	POSITIVE	POSITIVE	29.73	28.03	32.63	28.23	28.01	26.5
PAP0059	POSITIVE	POSITIVE	17.66	16.03	26.36	17.2	15.25	24.79

Brand C

CODE	Control swab	Test swab	C			T		
			N1	N2	RnaseP	N1	N2	RnaseP
MIT0001	POSITIVE	POSITIVE	19.22	17.49	26.66	22.61	21.43	27.04
MIT0002	NEGATIVE	INCONCLUSIVE	N/A	N/A	29.24	N/A	38.94	31.21
MIT0003	NEGATIVE	INCONCLUSIVE	N/A	N/A	35.82	N/A	37.48	29.87
MIT0007	POSITIVE	POSITIVE	24.36	25.15	28.82	28.57	28.36	34.43
MIT0008	POSITIVE	INCONCLUSIVE	36.71	32.32	27.04	N/A	35.51	35.51
MIT0010	POSITIVE	POSITIVE	27.95	28.57	28.57	28.23	28.38	28.38
MIT0015	POSITIVE	POSITIVE	22.96	21.78	29.21	21.01	20.35	28
MIT0017	POSITIVE	POSITIVE	17.94	16.19	16.19	21.35	20.34	20.34
MIT0018	INCONCLUSIVE	NEGATIVE	N/A	37.06	29.63	N/A	N/A	28.67
MIT0019	NEGATIVE	INCONCLUSIVE	N/A	41.63	30.09	N/A	37.48	28.17
MIT0021	POSITIVE	POSITIVE	30.93	30.17	28.69	32.44	31.14	27.17
MIT0022	POSITIVE	POSITIVE	17.53	16.22	27.47	20.29	18.48	29.62
MIT0023	INCONCLUSIVE	NEGATIVE	N/A	37.06	27.78	N/A	40.06	26.65
MIT0024	NEGATIVE	POSITIVE	N/A	N/A	25.54	38.85	34.62	24.76
MIT0025	POSITIVE	POSITIVE	19.03	17.88	25.9	22.02	20.3	26.08
MIT0026	NEGATIVE	INCONCLUSIVE	N/A	N/A	26.63	N/A	37.19	26.93
MIT0028	NEGATIVE	INCONCLUSIVE	N/A	N/A	26.93	N/A	33.82	28.94
MIT0033	INCONCLUSIVE	NEGATIVE	N/A	38.65	27.37	N/A	N/A	26.64
MIT0034	NEGATIVE	INCONCLUSIVE	N/A	N/A	25.88	N/A	39.1	25.37
MIT0035	POSITIVE	POSITIVE	20.14	20.07	27.18	33.47	33.59	29.07
MIT0040	NEGATIVE	INCONCLUSIVE	N/A	N/A	27.01	N/A	38.51	28.79
MIT0041	POSITIVE	POSITIVE	22.06	20.95	25.36	24.08	23.14	29.19
MIT0042	NEGATIVE	INCONCLUSIVE	N/A	41.76	26.43	N/A	38.78	30.58
MIT0056	POSITIVE	POSITIVE	16.89	15.75	27.78	14.72	13.31	25.99
MIT0057	POSITIVE	INCONCLUSIVE	37.1	33.52	27.4	N/A	38.71	30.84
MIT0058	POSITIVE	POSITIVE	31.93	31.28	27.8	20.91	19.6	26.21
MIT0059	INCONCLUSIVE	INCONCLUSIVE	N/A	40.3	31.1	N/A	37.73	26.35

MIT0061	POSITIVE	POSITIVE	18.84	18.43	28.83	12.1	11.38	25.82
MIT0062	POSITIVE	POSITIVE	35.37	34.64	29.11	36.46	37.13	30.49
MIT0063	POSITIVE	POSITIVE	15.29	13.77	25.74	19.08	17.01	28.12
MIT0064	POSITIVE	POSITIVE	38.15	37.4	28.97	38.27	36.35	29.25
MIT0066	POSITIVE	POSITIVE	17.5	17.66	27.34	20.05	19.15	28.07
MIT0073	NEGATIVE	INCONCLUSIVE	N/A	41.85	29.32	N/A	37.49	28.95
MIT0089	POSITIVE	POSITIVE	22.06	20.73	28.69	22.05	21.09	28.68
MIT0092	POSITIVE	POSITIVE	22.21	22.89	27.49	21.58	22.56	28.01
MIT0096	POSITIVE	POSITIVE	18.39	18.48	27.31	18.18	18.44	27.22
MIT0097	POSITIVE	POSITIVE	30.17	31.06	26.57	28.73	30.02	23.43
MIT0100	POSITIVE	POSITIVE	17.45	16.63	25.07	16.55	15.92	25.54
MIT0101	POSITIVE	POSITIVE	39.78	35.05	26.15	35.62	35.73	24.89
MIT0102	POSITIVE	POSITIVE	28.32	27.77	28.12	32.58	32.22	24.99
MIT0104	POSITIVE	POSITIVE	15.25	15.59	27.44	14.2	14.28	26.17
MIT0105	POSITIVE	POSITIVE	39.9	36.84	29.15	39.7	35.65	26.14
MIT0106	INCONCLUSIVE	POSITIVE	N/A	35.62	25.77	35.13	33.56	24.33
MIT0112	POSITIVE	POSITIVE	19.24	20	27.32	22.05	21.84	26.24
MIT0113	POSITIVE	POSITIVE	31.62	31.94	27.2	39.92	35.48	26.09
MIT0114	POSITIVE	POSITIVE	17.98	17.97	26.37	22.3	22.07	27.68

Brand D

CODE	Control swab	Test swab	C			T		
			N1	N2	RnaseP	N1	N2	RnaseP
STM0004	POSITIVE	POSITIVE	15.48	14.53	28.33	24.53	23.75	27.29
STM0005	POSITIVE	POSITIVE	18.86	17.78	30.39	18.56	17.68	27.44
STM0006	POSITIVE	NEGATIVE	21.66	21.29	25.89	N/A	N/A	27.47
STM0011	POSITIVE	POSITIVE	20.12	19.31	28.55	28.81	28.30	28.98
STM0013	POSITIVE	POSITIVE	31.88	32.11	28.95	28.44	28.48	29.68
STM0014	POSITIVE	POSITIVE	22.49	21.41	27.98	22.58	21.55	27.31
STM0015	POSITIVE	POSITIVE	26.29	25.20	30.06	24.06	23.07	25.4
STM0017	POSITIVE	POSITIVE	20.30	19.22	30.27	21.24	20.25	25.4
STM0018	POSITIVE	POSITIVE	28.47	27.93	29	24.59	23.82	27.13
STM0022	POSITIVE	POSITIVE	20.31	19.64	26.91	18.25	17.97	26.9
STM0024	POSITIVE	POSITIVE	19.55	19.20	30.01	23.92	23.32	31.12
STM0027	POSITIVE	POSITIVE	16.57	17.08	25.79	18.48	19.06	25.1
STM0028	POSITIVE	POSITIVE	37.42	34.68	29.13	29.41	29.32	30.03
STM0029	POSITIVE	POSITIVE	20.96	19.94	26.06	21.17	20.37	24.13
STM0030	POSITIVE	POSITIVE	22.97	22.60	27.46	25.50	24.86	25.02
STM0031	POSITIVE	POSITIVE	18.76	18.53	27.69	27.62	27.44	27.58
STM0032	POSITIVE	POSITIVE	22.69	22.39	30.23	26.63	26.16	28.81
STM0033	POSITIVE	POSITIVE	21.09	20.68	29.63	21.53	20.54	27.7
STM0035	POSITIVE	POSITIVE	32.60	32.01	29.01	35.41	33.73	27.39
STM0038	POSITIVE	POSITIVE	15.19	14.87	29.82	17.08	16.80	29.86
STM0040	POSITIVE	POSITIVE	26.58	26.16	29.88	22.02	21.55	29.66

STM0041	POSITIVE	POSITIVE	17.69	16.27	30.29	18.92	17.56	32.02
STM0042	POSITIVE	INCONCLUSIVE	33.85	34.11	N/A	N/A	34.63	37.96
STM0043	POSITIVE	POSITIVE	19.04	17.08	31.38	18.04	16.30	29.09
STM0044	POSITIVE	NEGATIVE	33.02	30.73	27.2	N/A	N/A	27.11
STM0047	POSITIVE	POSITIVE	14.56	13.65	26.69	13.84	12.17	26.75
STM0048	POSITIVE	POSITIVE	29.01	29.09	26.93	25.07	24.05	26.08
STM0049	POSITIVE	POSITIVE	35.55	33.31	28.92	33.78	32.03	26.38
STM0054	POSITIVE	POSITIVE	21.34	20.50	29.48	18.36	17.24	26.35
STM0059	POSITIVE	POSITIVE	17.04	16.05	28.38	16.06	15.12	27.68
STM0060	POSITIVE	POSITIVE	19.02	17.82	27.35	21.71	20.73	28.56

Brand E

CODE	Control swab	Test swab	C			T		
			N1	N2	RnaseP	N1	N2	RnaseP
TNJ0001	INCONCLUSIVE	POSITIVE	N/A	33.67	27.82	38.66	34.45	27.85
TNJ0002	POSITIVE	POSITIVE	37.76	34.09	28.37	38.7	36.01	26.69
TNJ0003	POSITIVE	INCONCLUSIVE	39.76	35.4	29.42	N/A	36.08	27.03
TNJ0004	INCONCLUSIVE	NEGATIVE	N/A	38.01	31.03	N/A	N/A	29.22
TNJ0006	POSITIVE	POSITIVE	20.68	19.66	28.2	23.1	21.51	26.47
TNJ0011	POSITIVE	POSITIVE	37.57	34.95	26.02	38.42	35.57	27.28
TNJ0012	POSITIVE	POSITIVE	31.12	31.21	30.06	21.72	20.17	28.02
TNJ0013	POSITIVE	POSITIVE	26.28	24.16	32.28	36.61	32.74	30.07
TNJ0014	POSITIVE	POSITIVE	22.06	20.53	30.1	19.94	18.49	25.94
TNJ0015	POSITIVE	POSITIVE	35.51	33.59	30.21	33.85	31.89	25.72
TNJ0017	INCONCLUSIVE	NEGATIVE	N/A	38.25	35.59	N/A	42.26	32.66

TNJ0019	NEGATIVE	INCONCLUSIVE	N/A	N/A	38.92	N/A	38.5	34.71
TNJ0021	POSITIVE	POSITIVE	22.29	21.05	30.89	15.86	15.05	25.11
TNJ0026	POSITIVE	POSITIVE	21.37	19.7	28.17	24.1	23.07	28.26
TNJ0036	NEGATIVE	POSITIVE	N/A	N/A	31.21	39.59	34.7	26.27
TNJ0038	POSITIVE	POSITIVE	25.13	25.06	27.17	23.03	22.73	28.2
TNJ0039	POSITIVE	POSITIVE	17.73	17	28.05	16.5	16.2	27.43
TNJ0040	POSITIVE	POSITIVE	22.12	20.97	27.29	18.72	17.58	28.28
TNJ0042	INCONCLUSIVE	NEGATIVE	N/A	37.25	26.84	N/A	N/A	29.1
TNJ0043	POSITIVE	POSITIVE	25.17	25.51	25.82	27.78	28.09	26.37
TNJ0045	INCONCLUSIVE	NEGATIVE	N/A	36.01	29.29	34.65	32.08	26.57
TNJ0046	POSITIVE	POSITIVE	20.97	20.06	28.66	23.54	22.74	27.17
TNJ0047	POSITIVE	POSITIVE	29.71	28.52	26.73	32.72	32.29	26.62
TNJ0052	POSITIVE	POSITIVE	20.51	20.97	29.67	21.36	21.79	27.49
TNJ0053	POSITIVE	POSITIVE	27.65	28.34	27.47	29.39	30.36	28.33
TNJ0057	NEGATIVE	POSITIVE	N/A	N/A	29.14	31.83	32.9	25.74
TNJ0058	POSITIVE	POSITIVE	20.03	20.01	27.54	24.85	24.22	27.31
TNJ0063	POSITIVE	POSITIVE	23.72	22.72	28.47	17.59	16.56	27.76
TNJ0065	POSITIVE	POSITIVE	19.72	19.43	26.09	18.87	18.55	26.99
TNJ0067	POSITIVE	POSITIVE	19.37	17.86	27.08	18.51	17.16	26.24
TNJ0070	POSITIVE	POSITIVE	37.67	38.92	27.74	39.49	39.43	26.37
TNJ0079	POSITIVE	POSITIVE	21.02	19.59	28.03	22.13	20.78	31.37
TNJ0080	POSITIVE	POSITIVE	14.19	12.65	25.53	17.14	15.69	26.24
TNJ0081	INCONCLUSIVE	POSITIVE	N/A	38.76	27.61	39.81	35.02	25.98
TNJ0084	POSITIVE	POSITIVE	17.43	16.74	25.41	19.35	18.65	27.45
TNJ0095	NEGATIVE	POSITIVE	N/A	N/A	29.04	39.5	38.3	28.69
TNJ0097	POSITIVE	POSITIVE	17.22	17.29	26.99	16.46	16.62	26.55
TNJ0098	POSITIVE	POSITIVE	14.15	14.34	26.64	15.17	15.26	27.25
TNJ0099	NEGATIVE	POSITIVE	N/A	N/A	29.05	31.99	32.38	28.26
TNJ0100	POSITIVE	POSITIVE	32.45	32.71	30.46	25.84	25.07	26.75
TNJ0101	POSITIVE	POSITIVE	18.01	17.19	29.07	23.41	22.46	27.55

Brand F

CODE	Control swab	Test swab	C			T		
			N1	N2	RnaseP	N1	N2	RnaseP
PRI0001	POSITIVE	POSITIVE	20.07	19.6	28.07	16.13	16.01	24.21
PRI0002	POSITIVE	POSITIVE	18.22	17.9	30.09	15.77	15.24	26.03
PRI0003	POSITIVE	POSITIVE	37.37	34.46	39.55	29.32	28.45	26.33
PRI0004	INCONCLUSIVE	POSITIVE	N/A	36.17	29.64	39.49	38.42	24.65
PRI0005	INCONCLUSIVE	POSITIVE	N/A	37.36	28.93	36.05	33.57	24.74
PRI0010	POSITIVE	NEGATIVO	36.83	35.02	28.09	N/A	29.91	29.91
PRI0012	POSITIVE	POSITIVE	22.64	22.29	26.87	21.22	22.29	26.87
PRI0015	NEGATIVE	INCONCLUSIVE	N/A	N/A	34	N/A	38.36	25.66
PRI0016	POSITIVE	POSITIVE	33.25	25.54	26.95	25.31	24.29	24.94
PRI0017	POSITIVE	POSITIVE	25.91	25.27	28.13	20.04	18.23	26.86
PRI0020	POSITIVE	POSITIVE	16.81	15.04	26.02	20.02	18.19	26.02
PRI0021	INCONCLUSIVE	POSITIVE	N/A	34.63	25.66	38.7	33.85	25.27
PRI0023	INCONCLUSIVE	POSITIVE	N/A	38.03	27.67	35.47	34.5	26.06
PRI0028	POSITIVE	POSITIVE	18.98	17.04	26.02	23.77	22.45	25.47
PRI0029	POSITIVE	POSITIVE	21.25	21.06	27.98	19.15	18.37	25.23
PRI0030	POSITIVE	POSITIVE	15.46	15.02	27.56	12.15	11.7	24.67
PRI0031	POSITIVE	POSITIVE	19.64	18.06	26.44	20.34	19	26.1
PRI0033	POSITIVE	POSITIVE	34.68	32.26	28.18	27.11	26.32	25.12
PRI0034	POSITIVE	POSITIVE	36.96	33.5	26.57	35.61	33.64	25.83
PRI0035	POSITIVE	POSITIVE	39.62	34.08	27.07	36.78	36.04	24.68
PRI0036	POSITIVE	POSITIVE	30.55	30.51	24.62	25.49	23.73	23.85
PRI0037	POSITIVE	POSITIVE	17.89	15.95	25.51	17.32	14.71	24.82
PRI0038	INCONCLUSIVE	POSITIVE	N/A	36.97	27.16	37.29	35.67	25.43
PRI0039	INCONCLUSIVE	INCONCLUSIVE	N/A	37.77	28.72	N/A	36.75	25.74
PRI0040	POSITIVE	POSITIVE	32.8	32.05	28.41	32.58	32.06	29.02
PRI0043	POSITIVE	POSITIVE	18.1	16.49	27.46	17.49	15.92	26.27
PRI0045	NEGATIVE	POSITIVE	N/A	N/A	29.53	36.1	34.21	25.21
PRI0046	INCONCLUSIVE	POSITIVE	N/A	36.21	28.59	39.96	34.75	25.53

PRI0047	POSITIVE	POSITIVE	39.98	33.58	24.8	37.43	35.07	24.15
PRI0048	POSITIVE	POSITIVE	16.53	14.84	26.89	16.25	14.74	25.53
PRI0049	POSITIVE	POSITIVE	34.72	34.41	25.45	37.46	35.73	25.54
PRI0050	POSITIVE	POSITIVE	17.46	15.33	25.74	18.82	16.67	24.46
PRI0051	POSITIVE	POSITIVE	23.18	21.45	26.52	15.94	14.61	26.1
PRI0052	POSITIVE	POSITIVE	35.95	37.98	26.53	37.57	34.07	24.2
PRI0053	POSITIVE	POSITIVE	19.68	18.45	26.18	20.87	19.47	24.82
PRI0054	POSITIVE	NEGATIVE	39.94	36.19	26.46	N/A	N/A	25.97
PRI0055	POSITIVE	POSITIVE	38.5	35.02	26.16	37.42	34.9	25.47
PRI0056	POSITIVE	INCONCLUSIVE	39.92	35.63	30.06	N/A	37.53	27.3
PRI0057	POSITIVE	POSITIVE	N/A	40.49	29.49	39.73	36.42	29.21
PRI0058	POSITIVE	POSITIVE	22.64	20.07	25.88	22.15	19.76	24.86
PRI0059	POSITIVE	POSITIVE	30.08	28.75	26.24	23.03	22.68	26.79
PRI0060	INCONCLUSIVE	POSITIVE	N/A	35.83	28.95	32.21	35.16	27.07
PRI0061	POSITIVE	POSITIVE	29.27	28.75	33.74	21.94	20.61	29.24
PRI0063	POSITIVE	POSITIVE	32.1	31.65	29.48	31.49	31.36	25.89
PRI0064	POSITIVE	INCONCLUSIVE	37.66	35.37	28.47	N/A	35.24	24.13

Brand G

CODE	Control swab	Test swab	C			T		
			N1	N2	RnaseP	N1	N2	RnaseP
CHS0001	POSITIVE	POSITIVE	27.25	27.54	29.48	29.79	30.31	28.54
CHS0006	POSITIVE	POSITIVE	21.66	21.29	27.8	20.24	19.69	28.11
CHS0009	POSITIVE	POSITIVE	16.35	15.85	28.07	17.43	16.55	25.32
CHS0010	POSITIVE	POSITIVE	21.78	20.55	26.66	24.54	23.87	24.84
CHS0011	POSITIVE	POSITIVE	28.98	28.61	25.02	35.61	30.68	25.81
CHS0014	POSITIVE	POSITIVE	20.12	19.42	27.72	19.78	19.00	27.15
CHS0015	POSITIVE	POSITIVE	26.08	25.35	29.1	22.19	21.93	31.36

CHS0018	NEGATIVE	POSITIVE	N/A	40.01	33.01	24.51	24.56	31.72
CHS0021	POSITIVE	POSITIVE	15.36	16.23	29.75	17.05	17.55	31.52
CHS0022	POSITIVE	POSITIVE	16.99	16.64	25.33	17.18	17.18	27.31
CHS0024	POSITIVE	POSITIVE	20.21	19.62	29.71	21.06	21.02	28.3
CHS0025	POSITIVE	POSITIVE	26.31	26.10	30.93	30.28	31.04	31.7
CHS0026	POSITIVE	POSITIVE	15.74	15.52	26.45	18.63	18.35	30.04
CHS0031	POSITIVE	POSITIVE	31.95	32.43	29.77	39.71	34.80	30.49
CHS0032	POSITIVE	POSITIVE	15.28	15.96	27.82	19.56	20.11	30.59
CHS0033	POSITIVE	POSITIVE	20.05	19.52	28.48	18.56	18.05	30.02
CHS0036	POSITIVE	POSITIVE	24.97	25.45	30.38	23.18	23.80	29.62
CHS0037	POSITIVE	POSITIVE	22.14	22.25	30.92	19.17	19.11	29.81
CHS0038	POSITIVE	POSITIVE	30.43	30.56	29.79	21.33	20.97	27.73
CHS0040	POSITIVE	POSITIVE	29.01	29.30	31.13	27.30	27.49	30.38
CHS0041	POSITIVE	POSITIVE	18.71	18.27	26.97	20.37	19.83	28.19
CHS0043	POSITIVE	POSITIVE	28.51	28.59	31.02	31.02	31.25	30.1
CHS0044	POSITIVE	POSITIVE	17.91	17.36	27.74	27.74	19.45	29.04
CHS0045	POSITIVE	POSITIVE	27.22	26.94	30.08	30.08	24.19	30.03
CHS0046	POSITIVE	POSITIVE	15.18	14.77	26.48	26.48	15.53	27.97
CHS0047	POSITIVE	POSITIVE	17.90	17.04	29.93	17.01	16.46	30.29
CHS0049	POSITIVE	POSITIVE	24.58	24.01	29.1	34.28	33.96	30.17
CHS0051	POSITIVE	POSITIVE	20.00	19.38	29.62	20.46	20.06	29
CHS0052	POSITIVE	POSITIVE	35.70	35.30	33.33	34.43	33.53	32.77
CHS0053	POSITIVE	POSITIVE	15.89	16.02	29.08	17.57	17.43	29.13
CHS0056	NEGATIVE	POSITIVE	N/A	N/A	39.75	37.76	36.16	N/A
CHS0057	POSITIVE	POSITIVE	18.02	16.41	28.72	17.39	16.17	23.44
CHS0058	INCONCLUSIVE	NEGATIVE	N/A	34.65	29.03	N/A	41.65	30.1
CHS0059	POSITIVE	POSITIVE	18.81	18.04	28.82	22.14	21.04	28.15
CHS0060	POSITIVE	POSITIVE	23.23	22.06	28.02	23.11	22.40	29.09
CHS0061	NEGATIVE	INCONCLUSIVE	N/A	42.15	29.09	N/A	37.91	28.07
CHS0062	POSITIVE	POSITIVE	21.00	19.74	28.22	18.39	17.18	28.71
