# Supplementary figures and images for: Original article: novelty of Canadian manufacture nasopharyngeal swabs for collection of samples being tested for SARS-CoV-2 in a pandemic setting
Source: Front Public Health. 2024 May 9;12:1344295. doi: 10.3389/fpubh.2024.1344295 (PMC11111943; doi:10.3389/fpubh.2024.1344295)

Supplementary Figure 1a: Testing Flow Chart

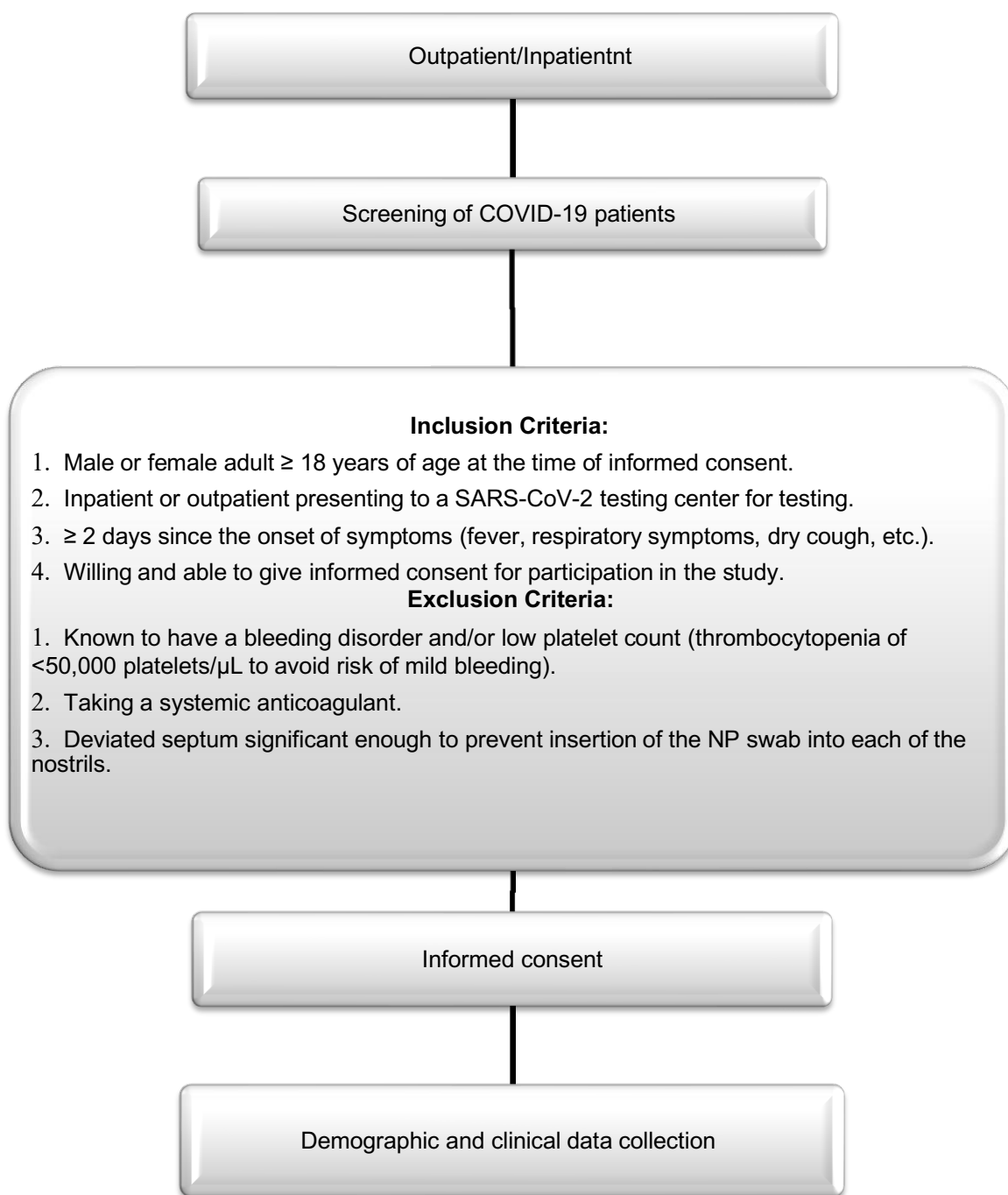

Supplement: Supplementary file 2 [file Image_1.pdf]

Supplementary Figure 1b: Testing Flow Chart

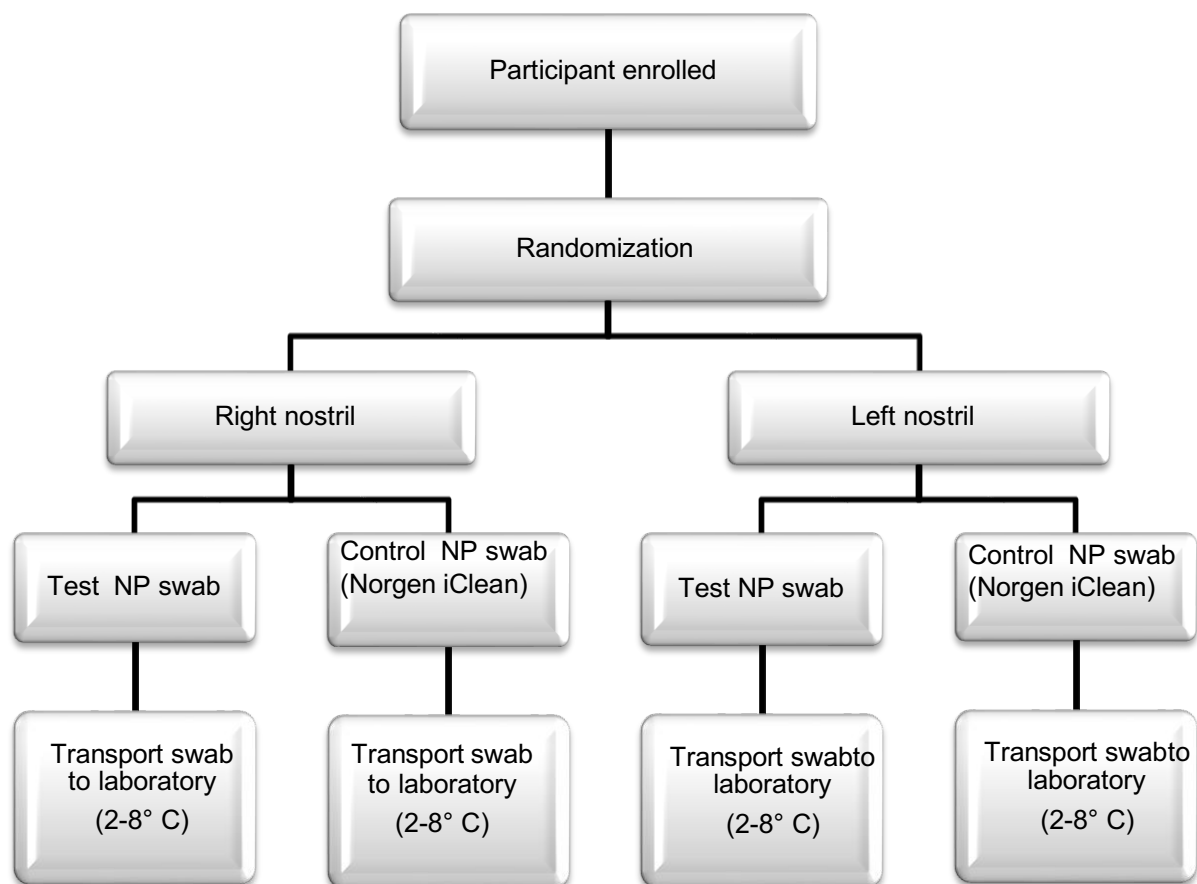

Supplement: Supplementary file 3 [file Image_2.pdf]

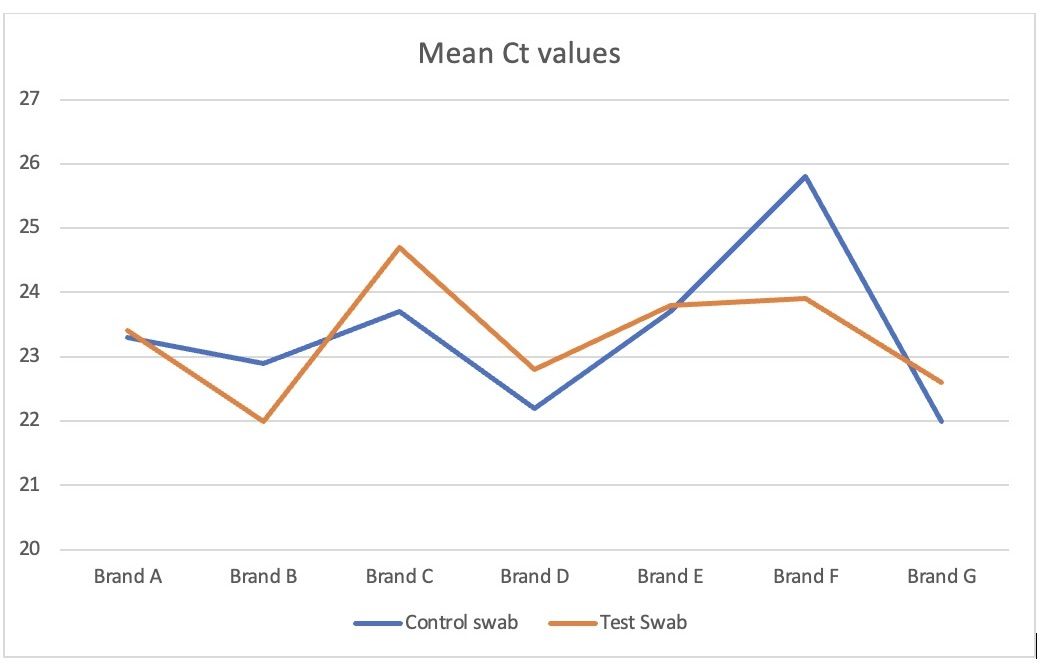

Supplement: Supplementary file 4 [file Image_3.tiff]
